# Evidence of Leptospiral Presence in the Cumberland Gap Region

**DOI:** 10.1371/journal.pntd.0007990

**Published:** 2019-12-26

**Authors:** Ashutosh Verma, Brittney Beigel, Christopher Carl Smola, Susanna Kitts-Morgan, Daniel Kish, Paul Nader, Joey Morgan, Jerry Roberson, Undine Christmann, Karen Gruszynski, LaRoy Brandt, Ellen Cho, Kelly Murphy, Ryan Goss

**Affiliations:** 1 Center for Infectious, Zoonotic and Vector-borne diseases, Lincoln Memorial University, Harrogate, Tennessee, United States of America; 2 College of Veterinary Medicine, Lincoln Memorial University, Harrogate, Tennessee, United States of America; 3 Center for Animal and Human Health in Appalachia, Lincoln Memorial University, Harrogate, Tennessee, United States of America; 4 Cumberland Mountain Research Center, Lincoln Memorial University, Harrogate, Tennessee, United States of America; 5 School of Mathematics and Science, Lincoln Memorial University, Harrogate, Tennessee, United States of America; Baylor College of Medicine, UNITED STATES

## Abstract

**Background:**

Leptospirosis is a widespread zoonotic disease that causes reproductive losses and/or hepatorenal failure in a number of animal species. Wild reservoirs of the disease, such as rodents, harbor the causative bacterium, *Leptospira* spp., in their kidneys and contaminate the environment by excreting infected urine. In this study, we tested small wild mammals, environmental water, and livestock in the Cumberland Gap region of southeastern Appalachia for the presence of pathogenic *Leptospira* or leptospiral antibodies.

**Methods/Results:**

Small wild mammals (n = 101) and environmental water samples (n = 89) were screened by a real time quantitative PCR that targets the pathogenic *Leptospira*-specific *lipl32* gene. Kidneys from 63 small wild mammals (62.37%) and two water sources (2.25%) tested positive for leptospiral DNA. To identify the infecting leptospiral species in qPCR-positive water and kidney samples, a fragment of leptospiral *rpoB* gene was PCR amplified and sequenced. *L*. *kirschneri* and *L*. *interrogans* were the leptospiral species carried by small wild mammals. Furthermore, sera from livestock (n = 52; cattle and horses) were screened for leptospiral antibodies using microscopic agglutination test (MAT). Twenty sera (38.46%) from livestock had antibodies to one or more serovars of pathogenic *Leptospira* spp.

**Conclusions:**

In conclusion, results from our study show exposure to leptospiral infection in farm animals and the presence of this zoonotic pathogen in the environmental water and kidneys of a significant number of small wild mammals. The public health implications of these findings remain to be assessed.

## Author summary

Leptospirosis, a zoonotic disease caused by *Leptospira*, affects people and animals, including dogs, cows and horses. Many species of small mammals, and particularly rodents, are believed to be natural reservoirs of the pathogen across numerous geographical ranges with similar climatic conditions. The Cumberland Gap region (CGR) in southern Appalachia has several climatic risk factors for leptospirosis. With this in mind, we tested surface water, kidneys of small wild mammals, and blood from cows and horses in the CGR for the presence of leptospiral DNA or antibodies. Notably, more than 60% of tested small mammals carried leptospires in their kidneys. Furthermore, we found that 2 of the 89 tested environmental water samples contained leptospiral DNA, and a significant percentage of cows and horses had leptospiral antibodies. These findings will advance our understanding of the ecology of leptospirosis in the region, which will lead to stakeholder awareness, and development of preventive interventions with broad applicability within and outside this region of southern Appalachia.

## Introduction

Leptospirosis is a worldwide veterinary and public health problem caused by pathogenic spirochetes of the genus *Leptospira* [1,2]. Pathogenic leptospires have been detected in over 150 mammalian species and the global burden of the disease is estimated to be approximately 1 million cases per year [3,4,5]. The spectrum of clinical presentations in human leptospirosis ranges from a mild flu-like form to a potentially fatal syndrome involving multi-organ failure. Leptospiral infection in domestic livestock results in significant losses due to spontaneous abortion, infertility, lowered milk production, and death [6].

The disease is maintained in the environment due to chronic renal infection of domestic carrier animals and wild reservoirs. Wild small mammals, especially rodents, play a particularly important role in the transmission cycle. These animals shed leptospires in their urine, thus contaminating the environment and exposing humans and other animals to the pathogen. Additionally, leptospirosis is an occupational threat to farmers, dairy workers, abattoir workers, meat inspectors, veterinarians, and landscaping and rodent control workers, who are routinely exposed to animals or stagnant/slow-moving surface water. Leptospirosis is also considered a recreational hazard for people who swim in contaminated water bodies [3,5,6].

Leptospirosis is widely distributed among different animal hosts in the United States. A leptospiral serosurveillance reported a prevalence of 45% among horse populations in 29 US states and one Canadian province [7]. Canine cases of leptospirosis in the US have also shown a steady increase over the years, with some areas being more affected than others [8,9,10]. A recent study on canine leptospirosis identified many Appalachian counties in Eastern Kentucky, Western Virginia, and West Virginia to have the highest overall predicted risk in the US [11].

The Cumberland Gap, located within the Cumberland Gap National Historical Park, is a natural throughway in the Southeastern Appalachian plateau close to the intersection of the state boundaries of Kentucky, Tennessee, and Virginia. The Cumberland Gap region (CGR) is primarily rural with hot and humid summers, mild winters, high annual precipitation, dense forest cover and low socioeconomic status of its residents. The CGR is also home to many species of small wild mammals, including rodents [12]. All the above-mentioned climatic, topographical, and socioeconomic factors have been described as risk predictors for the occurrence of leptospirosis [11].

Since no information was available regarding prevalence of leptospirosis in the CGR, we tested small wild mammals, environmental water, and livestock for the presence of pathogenic *Leptospira* or leptospiral antibodies. Environmental surface water and kidneys of small wild mammals were screened for the presence of leptospiral DNA using a highly sensitive and specific TaqMan-based qPCR. Additionally, horses and cattle were screened for the presence of leptospiral antibodies using microscopic agglutination test, a gold standard in leptospiral serodiagnosis.

## Materials and methods

### Study areas

**Fig 1:**
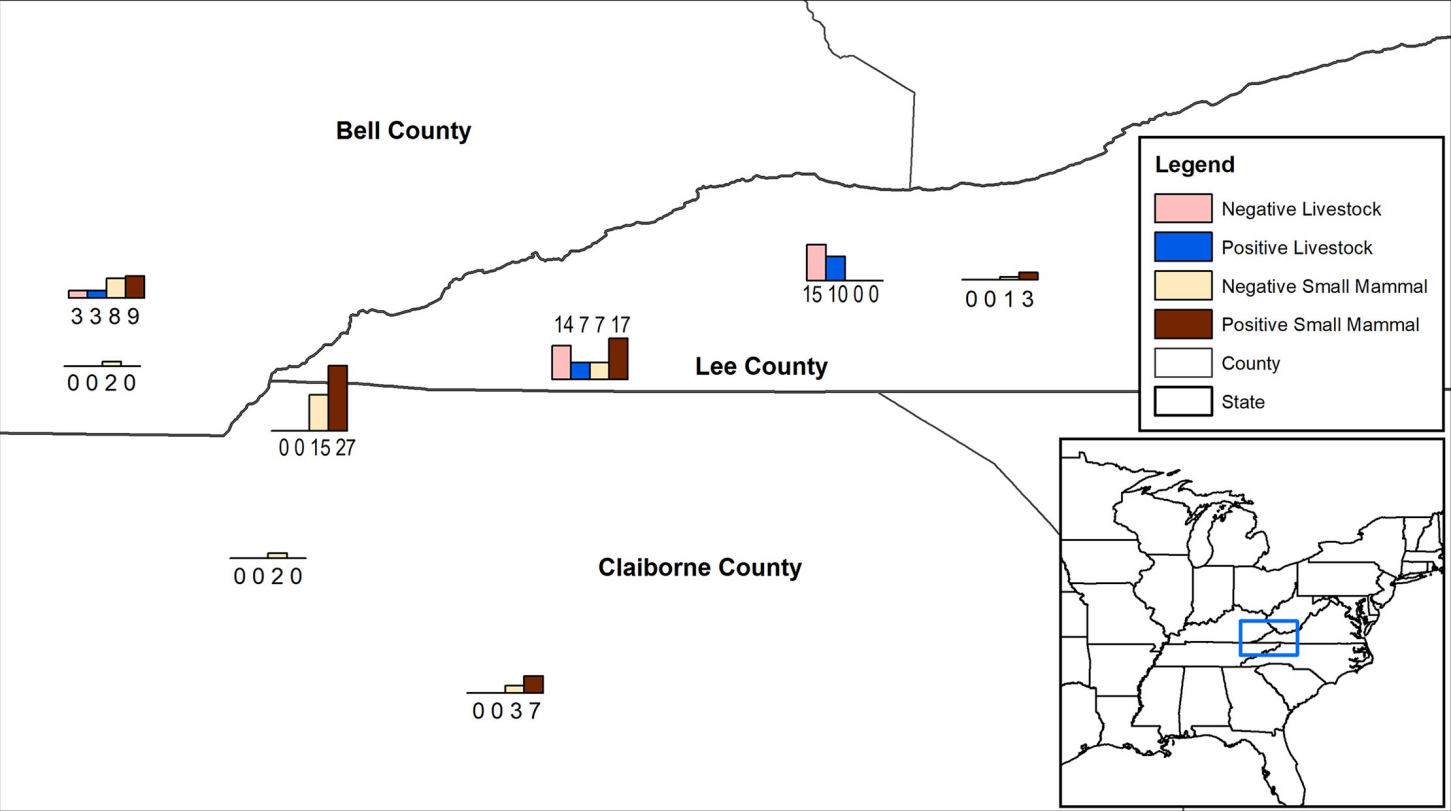
Map with histograms displaying the number of negative livestock, negative small mammals, positive livestock, and positive small mammals at different study sites located within the Cumberland Gap Region of Kentucky, Tennessee, and Virginia. Map created with ArcMap 10.6 (Esri, Redlands, CA).

**Fig 2:**
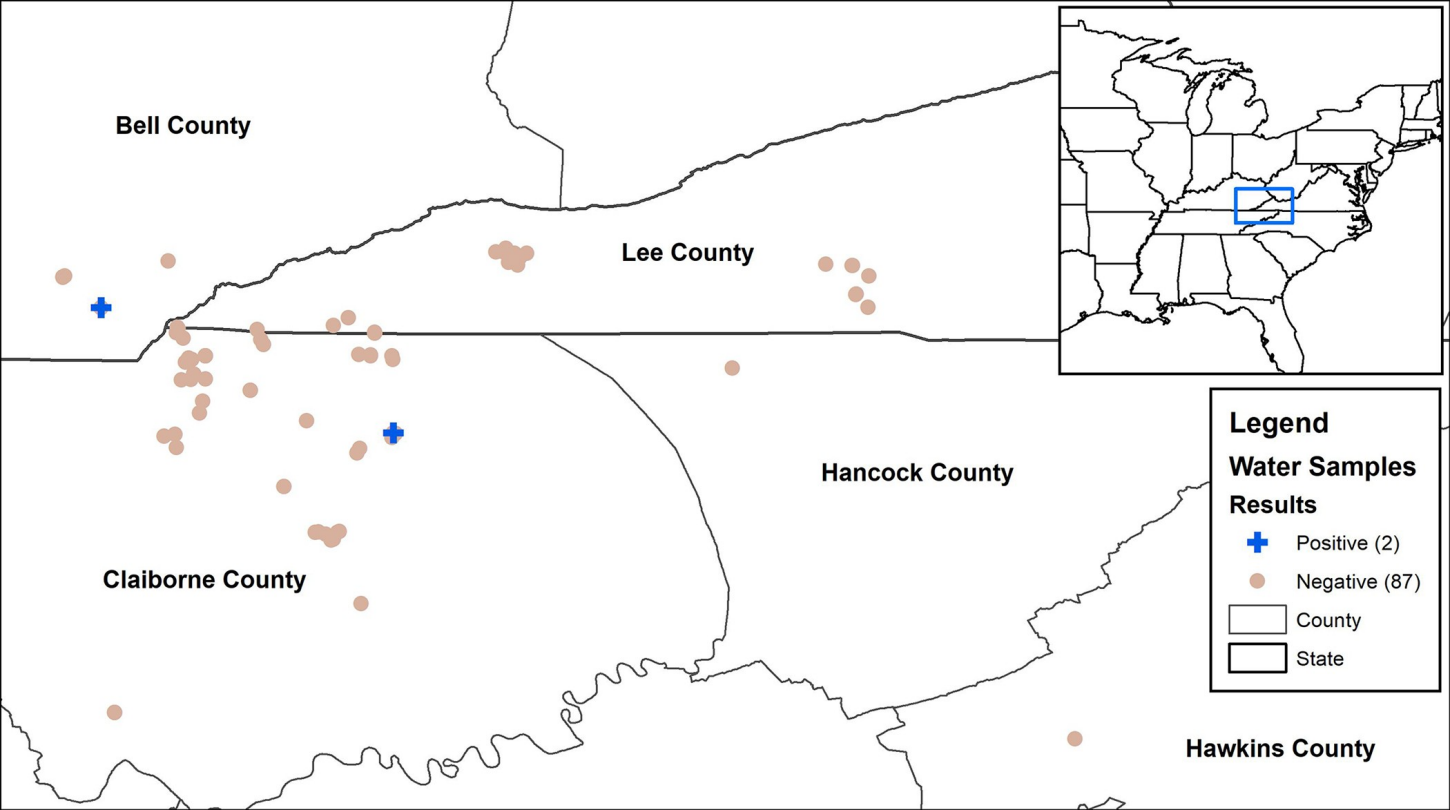
Map displaying the five counties in Kentucky, Tennessee, and Virginia where 89 water samples were taken for Leptospira qPCR testing. The two positive samples are displayed as blue plus signs. Map created with ArcMap 10.6 (Esri, Redlands, CA).

Water collection and small wild mammal trapping was performed primarily in Northeast Tennessee (Claiborne county), Southeast Kentucky (Bell county), and Southwest Virginia (Lee county) (**Fig 1**). Additional water samples were collected from Hawkins and Hancock counties in Tennessee (**Fig 2**). General topography in this area consists of mountains, rolling hills within open pastures, woods, small creeks and ponds. Areas included in this study were comprised of a combination of these features and various types of buildings (barns, sheds, and outbuildings).

### Small wild mammal trapping

Sherman live traps of various sizes ranging from small (233 mm L x 77 mm W x 88 mm H) to medium (301 mm L x 77 mm W x 88 mm H), to large (377 mm L x 103 mm W x 122 mm H) were used to trap small mammals from March–July, 2017. Traps were baited with a mixture of rolled and quick oats, plus walnuts. Traps were checked daily and endangered, threatened, or protected species were immediately released. After identification, species approved for euthanasia were placed in a plastic euthanasia chamber (42.7 cm L x 30.5 cm W x 17 cm H) with an attached hose to deliver CO_2_ at a flow rate of 4.4 L/min. Two minutes following breathing cessation, CO_2_ flow was discontinued and a bilateral thoracotomy was performed. Following death, the abdominal cavity was opened and each kidney removed using a sterile scalpel. Kidneys were immediately frozen at -80°C until further analysis.

### Water collection

Three hundred milliliters (300ml) of samples were collected from 89 open water sites on farms (ephemeral stream/ditches (20); ponds (n = 24); puddles (n = 10); slow-moving creeks (n = 23); spring water (n = 4); water tanks/reservoirs (n = 8)) in the three states from March 2016 –July 2017 (**Fig 2**). Each sample received a unique number and was stored at -20°C until further processed [13,14]. For DNA extraction, samples were thawed and centrifuged at 3000 g for 30 min at 6°C and pellets were processed as described below.

### DNA extraction

DNA was extracted from 20 mg of kidney tissue and 300 mL of water samples using DNeasy Blood and Tissue kit (Qiagen, Valencia, CA, USA). Water samples were centrifuged as described above and pellets were processed following manufacturer’s instructions with some modifications [15]. *Leptospira interrogans* serovar Pomona was grown in Polysorbate-80 bovine serum albumin medium (NVSL) at 30°C, and genomic DNA was extracted and quantified as previously described [16]. Based on the genome size of *L*. *interrogans* (4.659 Mb), genome equivalents were calculated [16].

### Quantitative Polymerase Chain Reaction (qPCR)

We used a TaqMan based quantitative PCR (qPCR) to target a 242 bp region of leptospiral *lipl32* gene, as previously described [17]. The assay was performed in a MicroAmp Fast Optical 96-well reaction plate (Applied Biosystems, Foster City, CA, USA). A standard curve was created using DNA standards equivalent to 10^7^, 10^6^, 10^5^, 10^4^, 10^3^, 10^2^, 10, 1 leptospiral genome units. Each column containing samples had a no-template control. Each reaction was performed in a 25 μL final volume, using 5 μL of extracted DNA, 500 nM of LipL32-45F (forward primer; 5ꞌ- AAGCATTACCG CTTGTGGTG-3ꞌ), 500 nM of LipL32-286R (reverse primer; 5ꞌ-GAACTCCCATTTCAGCGATT-3ꞌ) and 100 nM of LipL32-189P (probe; FAM-5ꞌ-AAAGCCAGGACAAGCGCCG-3ꞌ-BHQ1) [17]. The assay was performed on a QuantStudio 3 using Platinum Quantitative PCR SuperMix-UDG (Invitrogen, Carlsbad, CA, USA) and thermal conditions of a holding stage of 95°C for 20 s, and 40 cycles of 95°C for 3 s and 60°C for 30 s. All samples were run in duplicate and repeated at least twice.

### Livestock serum samples

Fifty-two blood samples were collected from horses (n = 31) and cattle (n = 21) farms in Virginia and Kentucky (**Fig 1**). Ten milliliters of blood were obtained from the jugular vein using a vacutainer needle (20G, 1.5”), vacutainer sleeve, and a 10 ml dry blood collection tube (red top). Clotted blood samples were centrifuged at 2,000 x g for 15 minutes. Serum was separated, stored frozen at -20°C, and when required, shipped on dry ice. None of the horses included in this study were vaccinated for leptospirosis. Vaccination history of cattle was not available, but the cattle farm practices periodic leptospiral vaccination of all its animals.

### Microscopic agglutination test

Microscopic agglutination test was performed at the University of Kentucky Veterinary Diagnostic Laboratory, an AAVLD accredited veterinary diagnostic lab, following OIE protocol (https://www.oie.int/fileadmin/Home/eng/Health_standards/tahm/3.01.12_LEPTO.pdf). Two-fold serum dilutions from 1:100 to 1:6400 were tested against serovars Canicola, Grippotyphosa, Icterohaemorrhagiae, and Pomona. The titer was defined as the reciprocal of the highest dilution of a serum sample that agglutinated more than half of leptospires. Titers of more than or equal to 1:100 were considered positive for the presence of leptospiral antibodies.

### Leptospiral *rpoB* gene sequencing

PCR amplification of a fragment of leptospiral *rpoB* gene and its sequencing was done for all the positive kidney and water samples as described previously [18]. Briefly, DNA from all qPCR-positive samples were subjected to PCR amplification of a 600bp fragment of *rpoB* gene using a Phusion High Fidelity polymerase (Thermofisher, Waltham, MA), primers Lept 1900f (5’-CCT-CAT-GGG-TTC-CAA-CAT-GCA-3’) and Lept 2500r (5’-CGC-ATC-CTC-RAA-GTT-GTA-WCC-TT-3’), and thermal conditions as described previously [18]. PCR amplicons were sequenced at a commercial sequencing facility (Davis sequencing, Davis, CA), and compared to available sequences by BLAST search using the National Center for Biotechnology Information server (http://www.ncbi.nlm.nih.gov/BLAST/).

### Ethics statement

All animal experiments were carried out in strict accordance with the recommendations in the Animal Welfare Act of 1966, its amendments and associated Regulations (https://www.nal.usda.gov/awic/animal-welfare-act). All protocols were reviewed and approved by the Animal Care and Use Committee at the Lincoln Memorial University (protocol numbers: 1602-CVM-05, 1815-CVM and 1819-CVM). Trapping of wild small mammals was conducted on Lincoln Memorial University property and private land. Permission was granted from all private landowners. No endangered, threatened, or protected species were euthanized in this study. Small mammals live-trapped and euthanized in this study were collected under the following state permits: Kentucky Department of Fish and Wildlife Resources, Educational Wildlife Collecting Permit #SC1711002, Virginia Department of Game and Inland Fisheries, Scientific Collection Permit #058453, and Tennessee Wildlife Resources Agency, Scientific Collection Permit #3954.

## Results

### Leptospiral carriage in small wild mammals

A total of 101 small wild mammals from CGR were screened for leptospiral presence in their kidneys by a TaqMan-based qPCR that targets *lipl32* gene. More than half of these animals (n = 54) were collected from Claiborne county, TN, followed by Lee county, VA (n = 28), and Bell county, KY (n = 12). Of 101 animals captured, ninety animals were rodents, which included deer mouse (*Peromyscus maniculatus*; n = 36), house mouse (*Mus musculus*; n = 28), Hispid cotton rat (*Sigmodon hispidus*; n = 25), and Eastern harvest mouse (*Reithrodontomys humulis*; n = 1). The remaining animals included shrews (n = 6), vole (n = 1), squirrel (n = 1), chipmunk (n = 2), and cottontail (n = 1) (**Table 1**).

**Table 1:** Leptospiral carriage in small wild mammal species.

Species Name	Number of Samples	Positive by qPCR (%)	*Average concentration (GE/gram of kidney)	^#^Standard deviation
Eastern gray squirrel (*Sciurus carolinensis*)	1	0 (0%)	-	-
Eastern chipmunk (*Tamias striatus*)	2	2 (100%)	1.9 x 10^4^	6.3 x 10^3^
Eastern cottontail (*Sylvilagus floridanus*)	1	0 (0%)	-	-
Eastern harvest mouse (*Reithrodontomys humulis*)	1	1 (100%)	8.3 x 10^3^	-
Hispid cotton rat (*Sigmodon hispidus*)	25	17 (68%)	3.7 x 10^7^	1.2 x 10^8^
House mouse (*Mus musculus*)	28	16 (54.17%)	4.8 x 10^6^	8.9 x 10^6^
Deer mouse (*Peromyscus maniculatus*)	36	23 (63.89%)	1.08 x 10^5^	2.4 x 10^5^
Northern American least shrew (*Cryptotis parva*)	2	1 (50%)	2.3 x 10^4^	-
Northern short-tailed shrew (*Blarina brevicauda*)	4	2 (50%)	4.1 x 10^4^	3.8 x 10^3^
Southern red-backed vole (*Myodes gapperi*)	1	1 (100%)	3.7 x 10^4^	-

* Average of leptospiral genomic equivalents (GE) per gram of kidney tissue, for each species of small wild mammals, as detected by qPCR

*#* variation in leptospiral GE concentrations among different animals within in a host species

For 101 small wild mammals, DNA was extracted from 202 kidneys and screened by *lipl32*-specific qPCR. In total, 101 kidneys representing 63 animals (62.37%; 95% CI: 52.9% - 71.8%) were positive for the presence of leptospiral DNA (**Table 2**). The average leptospiral concentration (genomic units/gram of kidney tissue) among rodents varied from 8.3 x 10^3^ in Eastern harvest mouse (*Reithrodontomys humulis*) to 3.7 x 10^7^ in Hispid cotton rat (*Sigmodon hispidus*). Among non-rodent small mammal species, the average concentration (genomic units/gram of kidney) ranged from 1.9 x 10^4^–4.1 x 10^4^. Leptospiral concentration among individual animals within a species varied considerably, as shown by large standard deviation values (**Table 1**).

**Table 2:** Prevalence of *Leptospira* spp in the small wild mammals and environmental water, and leptospiral exposure of livestock.

Sample Type	Positive	Number of Samples	Percent Positive	95% Confidence Interval	Test
Water	2	89	2.25%	0% - 5.3%	qPCR
Small Wild Mammals	63	101	62.37%	52.9% - 71.8%	qPCR
Livestock (Horses and Cattle)	20	52	38.46%	25.2% - 51.7%	MAT

Out of 63 positive animals, 38 animals were positive in both kidneys and 25 animals were unilaterally positive. *Leptospira*-positive animals included both rodents (57/90; 63.33%; 95% CI 53.3% - 73.3%) and non-rodent small mammal species (6/11; 54.55%; 95% CI 25.6% - 84.4%) (**Table 1**).

A 600bp fragment of leptospiral *rpoB* gene was PCR amplified from DNA of qPCR-positive kidneys and sequenced. Twenty-two kidneys representing 12 small wild mammals yielded good quality *rpoB* gene sequences. Analyses of these sequences revealed >99% homology with *rpoB* gene fragments of *L*. *kirschneri* (16 kidneys representing 8 animals), and *L*. *interrogans* (6 kidneys representing 4 animals) homologous gene fragments. All animals (n = 8) positive for *L*. *kirschneri* were trapped on the same farm in Virginia. Similarly, 2 animals that tested positive for *L*. *interrogans* were trapped from a single residential location in Tennessee. *L*. *kirschneri* was present in the kidneys of *Mus musculus* (12 kidneys representing 6 animals), *Sigmodon hispidus* (2 kidneys representing one animal), and *Peromyscus maniculatus* (2 kidneys representing one animal). *L*. *interrogans* were present in two *Sigmodon hispidus* and two *Peromyscus maniculatus*. Nucleotide sequences deposited in the GenBank have accession numbers MN721845-MN721866 (**Supporting S1 Table**).

### Leptospiral contamination of environmental water

Next, we tested environmental water samples for the presence of leptospiral DNA using a *lipl32*- specific qPCR. This qPCR is important in screening environmental samples as it is specific for pathogenic serovars and does not detect saprophytic leptospires that may be present in the environment samples [19]. Eighty-nine water samples were collected from open water sources on agricultural and animal farms in five counties in Kentucky, Tennessee, and Virginia (**Fig 2**). Two samples (2.25%; 95% CI: 0% - 5.3%) were positive for leptospiral DNA by qPCR (**Table 2 and Fig 2**). One of the positive water samples was collected from Bell county, KY, and the other was procured from Claiborne county, TN. The two positive water samples from KY and TN contained 6.6 x 10^4^ and 2.7 x 10^5^ genome units/liter of water, respectively. *rpoB* gene fragment was PCR amplified from DNA of two qPCR-positive water samples and sequenced. Analyses of sequence revealed >99% homology with the *L*. *interrogans* homologous gene fragment. Nucleotide sequences deposited in the GenBank have accession numbers MN721867 and MN721868 (**Supporting S1 Table**)

### Leptospiral seropositivity in livestock

Microscopic agglutination test was performed to detect leptospiral antibodies in serum samples from horses and cattle. Fifty-two blood samples were drawn from horses (n = 31) and cattle (n = 21) in Virginia and Kentucky (**Fig 1**). Sera were separated and screened against leptospiral serovars Canicola, Grippotyphosa, Icterohaemorrhagiae, and Pomona. A total of 20 farm animals (38.46%; 95% CI: 25.2% - 51.7%) were positive for at least one serovar (**Table 2**). Of these 20 MAT-positive animals, 13 (41.9%; 95% CI: 24.5% - 59.8%) were horses and 7 (33.3%; 95% CI: 13.1% - 53.5%) were cattle. All seven MAT-positive cattle had antibodies to serovar Pomona. In addition to Pomona, four cattle also had antibodies to Canicola (n = 1), Icterohaemorrhagiae (n = 1) or Grippotyphosa (n = 2) (**Table 3**). Eleven of 13 MAT-positive horses had antibodies to Icterohaemorrhagiae (n = 4) or Pomona (n = 1) or both (n = 6). One of the six horses that had antibodies to Pomona and Icterohaemorrhagiae, also had antibodies to Canicola. Reactivity to serovar Grippotyphosa was seen in two equine sera (**Table 3**). Ten of 20 MAT-positive cattle and horses had a titer of ≥ 1:400 for at least one tested serovar (**Table 3**).

**Table 3:** Microscopic agglutination test (MAT)—Seropositivity in horse* and cattle^#^.

Titer	Canicola	Grippotyphosa	Icterohaemorrhagiae	Pomona
1:100		3	4	3
1:200	1	1	5	2
1:400	1		2	3
1:800				3
1:1600				1
1:3200				1

* Horses with a titer of >100: 41.9% (13/31; 95% CI:24.5% - 59.8%)

# Cattle with a titer of >100: 33.3% (7/21; 95% CI: 13.1% - 53.5%)

## Discussion

In the primary health care settings, the World Health Organization and International Leptospirosis Society recommend that antibiotic treatment for leptospirosis should start on the sole basis of epidemiological and clinical suspicion [20]. Since the clinical presentation in leptospirosis is nonspecific, understanding of epidemiology of the disease at the local level becomes critical. This study addresses some key components to understand the epidemiology of leptospirosis in the CGR, for example, reservoir animals, environmental contamination, infecting leptospiral species, and infection in farm animals.

In this study, more than 60% of small wild mammals trapped in this study had leptospires in their kidneys. Although 90% of small wild mammals trapped in the present study were rodents (*Peromyscus maniculatus*, *Mus musculus*, *Sigmodon hispidus*, and *Reithrodontomys humulis*), our results show that not only rodents, but also shrews, voles, and chipmunks are reservoirs of *Leptospira* spp. in this region. Prevalence of leptospiral renal carriage among small wild mammals can vary depending on the region and reservoir species. In a recent study from the city of San Juan, Puerto Rico, 61.1% of trapped rodents (n = 18) were found to carry pathogenic leptospires [21]. In another study, 40.6% of 35 tested wild mammals (*Cerdocyon thous*, *Nasua nasua*, *Ozotoceros bezoarticus*, and *Sus scrofa*) from the Pantanal biome of Brazil, shed leptospires in their urine [22]. On the Caribbean island of St. Kitts, 6.16% of mongooses were found to carry leptospires in their kidneys [23]. A study from Canada on leptospiral carriage in Norway rats showed an overall prevalence of 11.1% (n = 592) [24]. In two other studies on leptospiral infection of Norway rats, 21% were found positive in Germany and 34.7% in France [25,26].

The high prevalence of leptospires in the kidneys of small wild mammals in the CGR makes them a likely source of environmental contamination and exposure of farm animals to leptospiral infection. Favorable environmental conditions aid survival of leptospires for prolonged periods, which increases the risk of transmission to people and animals that come in contact with contaminated water or soil [27,28]. Warmer temperatures, high rainfall, and mild winters in this region may further aid in the survival and spread of the organism to susceptible hosts [2,11]. Twenty of 52 tested farm animals in this study were seropositive, with primary reactivity to serovar Pomona in cattle and serovars Pomona and Icterohaemorrhagiae in horses. Serovars Pomona and Icterohaemorrhagiae have previously been shown to cause infection in these two hosts in many parts of the world [29,30,31]. Of the 20 MAT-positive animals, 13 were horses and 7 were cattle. Although horses in this study did not receive any vaccination for leptospirosis, cattle were periodically vaccinated. It is thus likely that MAT titers in some of the cattle may be due to vaccination.

Partial *rpoB* gene sequencing is effective in identification of *Leptospira* spp. in clinical or environmental samples. Using this method, we found *L*. *kirschneri* and *L*. *interrogans* in kidneys of small wild mammals. *L*. *interrogans* and *L*. *kirschneri* are two of the most common leptospiral species present in rodents in Europe [32,33]. *L*. *interrogans* have also been reported from rodents in studies from Puerto Rico, Bangladesh and Thailand [21,34,35].

The TaqMan-based qPCR used in this study is a practical method for detecting leptospires in environmental samples due to its ability to detect a small number of genomic units and its specificity for pathogenic *Leptospira* spp. [15]. Both of these features are useful for effective screening of large water bodies that may contain saprophytic leptospires. Humans can be infected directly if they are occupationally exposed to infected urine or kidneys, but most of the human infections occur via indirect exposure to contaminated water or soil. In non-tropical countries, contact with contaminated water or soil has been shown as a frequent means of exposure. In Europe, more than half of human leptospirosis cases reported occupational or recreational exposure to water [36,37]. While the infectious dose of leptospires in humans is unknown, multiple incidents of infection acquired via recreational water exposure suggest that a low dose is sufficient to cause infection [38,39].

Earlier studies investigating leptospiral contamination of water in the South American and Caribbean regions found 13.5% of household and environmental water samples in Southern Chile, 36% and 34% of sewage and standing water in Brazil, 33.3–67.9% of samples in an urban-slum area in Peru and 20% of open water sources in St. Kitts to be positive for leptospiral DNA [15,40,41,42]. In this study, we found that 2 of the 89 tested open water sites were positive for leptospiral DNA. Our results from water sources essentially represent point prevalence of leptospiral DNA in the local environment. Future studies should focus on longitudinal monitoring of water and soil contamination, which is needed to fully understand the role of environmental exposure in maintenance of infection in the CGR.

In summary, this study is the first report on the presence of leptospires in small wild mammals in the Cumberland Gap region of Appalachia, along with information on environmental contamination, circulating leptospiral species, and serological evidence of exposure in livestock. These findings provide an important starting point for launching public health and environmental health interventions at the local level. Improving the awareness about the presence of the disease in the CGR could help in prevention and timely treatment of human cases of leptospirosis. Considering the climate, the natural environment, socioeconomic status, living conditions and other cultural drivers, further studies on leptospirosis in other animal species and regions in Appalachia should be considered.

## Supporting information

S1 Table GenBank accession numbers of leptospiral *rpoB* sequences amplified from positive small wild mammal kidneys and environmental water. (PPTX)

## References

[1] Bharti AR, Nally JE, Ricaldi JN, Matthias MA, Diaz MM, et al (2003) Leptospirosis: a zoonotic disease of global importance. Lancet Infect Dis 3: 757–771. 10.1016/s1473-3099(03)00830-2 14652202

[2] Levett PN (2001) Leptospirosis. Clin Microbiol Rev 14: 296–326. 10.1128/CMR.14.2.296-326.2001 11292640 PMC88975

[3] Ko AI, Goarant C, Picardeau M (2009) Leptospira: the dawn of the molecular genetics era for an emerging zoonotic pathogen. Nat Rev Microbiol 7: 736–747. 10.1038/nrmicro2208 19756012 PMC3384523

[4] Costa F, Hagan JE, Calcagno J, Kane M, Torgerson P, Martinez-Silveira MS, et al (2015) Global Morbidity and Mortality of Leptospirosis: A Systematic Review. PLoS Negl Trop Dis 9(9): e0003898 10.1371/journal.pntd.0003898 26379143 PMC4574773

[5] Adler B, de la Pena Moctezuma A (2010) Leptospira and leptospirosis. Vet Microbiol 140: 287–296. 10.1016/j.vetmic.2009.03.012 19345023

[6] Faine S, Adler B, Bolin C, Perolat P (1999) *Leptospira* and Leptospirosis, 2nd edition, MediSci.

[7] Carter CN, Cohen N, Steinman MN, Smith JL, Erol E, Brown S. Seroepidemiology of equine leptospirosis utilizing diagnostic laboratory specimens from 29 states (US) and one Canadian province. Proceedings of 55th Annual AAVLD Meet; p51.

[8] Ward MP, Glickman LT, Guptill LE (2002) Prevalence of and risk factors for leptospirosis among dogs in the United States and Canada: 677 cases (1970–1998). J Am Vet Med Assoc. 220(1):53–58. 10.2460/javma.2002.220.53 12680448

[9] Glickman LT, Moore GE, Glickman NW, Caldanaro RJ, Aucoin D, Lewis HB (2006) Purdue University-Banfield National Companion Animal Surveillance Program for emerging and zoonotic diseases. Vector Borne Zoonotic Dis. 6(1):14–23. 10.1089/vbz.2006.6.14 16584323

[10] Moore GE, Guptill LF, Glickman NW, Caldanaro RJ, Aucoin D, Glickman LT (2006) Canine leptospirosis, United States, 2002–2004. Emerg Infect Dis. 12(3):501–503. 10.3201/eid1203.050809 16704794 PMC3291439

[11] White AM, Zambrana-Torrelio C, Allen T, Rostal MK, Wright AK, Ball EC, Daszak P, Karesh WB (2017) Hotspots of canine leptospirosis in the United States of America. Vet J. 222:29–35. 10.1016/j.tvjl.2017.02.009 28410673

[12] http://www.tnwatchablewildlife.org.

[13] Vein J, Perrin A, Berny PJ, Benoit E, Leblond A, Kodjo A (2012) Adaptation of a real-time PCR method for the detection and quantification of pathogenic leptospires in environmental water. Can J Microbiol. 58(7):828–835. 10.1139/w2012-060 22698616

[14] Gilpin BJ Devane M, Nourozi F, Robson B, Scholes P, Lin S (2013) Recommendations for the processing and storage of water samples before polymerase chain reaction (PCR) analysis, New Zealand Journal of Marine and Freshwater Research, 47:4, 582–586,

[15] Rawlins J, Portanova A, Zuckerman I, Loftis A, Ceccato P, Willingham AL, Verma A (2014) Molecular detection of leptospiral DNA in environmental water on St. Kitts. Int J Environ Res Public Health. 11(8):7953–7960. 10.3390/ijerph110807953 25105546 PMC4143842

[16] Levett PN, Morey RE, Galloway RL, Turner DE, Steigerwalt AG, Mayer LW (2005) Detection of pathogenic leptospires by real-time quantitative PCR. J Med Microbiol. 54 (Pt 1):45–49. 10.1099/jmm.0.45860-0 15591254

[17] Stoddard RA, Gee JE, Wilkins PP, McCaustland K, Hoffmaster AR (2009) Detection of pathogenic Leptospira spp. through TaqMan polymerase chain reaction targeting the LipL32 gene. Diagn Microbiol Infect Dis. 64(3):247–255. 10.1016/j.diagmicrobio.2009.03.014 19395218

[18] La Scola B, Bui LT, Baranton G, Khamis A, Raoult D (2006) Partial rpoB gene sequencing for identification of Leptospira species. FEMS Microbiol Lett. 263(2):142–147. 10.1111/j.1574-6968.2006.00377.x 16978348

[19] Schneider AG, Casanovas-Massana A, Hacker KP, Wunder EA Jr, Begon M, Reis MG, Childs JE, Costa F, Lindow JC, Ko AI. (2018) Quantification of pathogenic Leptospira in the soils of a Brazilian urban slum. PLoS Negl Trop Dis. 12(4):e0006415 10.1371/journal.pntd.0006415 29624576 PMC5906024

[20] World Health Organization (2003) Human leptospirosis: Guidance for diagnosis, surveillance and control, World Health Organization.

[21] Briskin EA, Casanovas-Massana A, Ryff KR, Morales-Estrada S, Hamond C, Perez-Rodriguez NM, Benavidez KM, Weinberger DM, Castro-Arellano I, Wunder EA, Sharp TM, Rivera-Garcia B, Ko AI (2019) Seroprevalence, risk factors, and rodent reservoirs of leptospirosis in an urban community of Puerto Rico, 2015. J Infect Dis. pii: jiz339. 10.1093/infdis/jiz339 31342075 PMC6761939

[22] Vieira AS, Narduche L, Martins G, Schabib Péres IA, Zimmermann NP, Juliano RS, Pellegrin AO, Lilenbaum W (2016) Detection of wild animals as carriers of Leptospira by PCR in the Pantanal biome, Brazil. Acta Trop. 163:87–89. 10.1016/j.actatropica.2016.08.001 27496621

[23] Shiokawa K, Llanes A, Hindoyan A, Cruz-Martinez L, Welcome S, Rajeev S (2019) Peridomestic small Indian mongoose: An invasive species posing as potential zoonotic risk for leptospirosis in the Caribbean. Acta Trop. 190:166–170. 10.1016/j.actatropica.2018.11.019 30465742

[24] Himsworth CG, Bidulka J, Parsons KL, Feng AY, Tang P, Jardine CM, Kerr T, Mak S, Robinson J, Patrick DM (2013) Ecology of Leptospira interrogans in Norway rats (Rattus norvegicus) in an inner-city neighborhood of Vancouver, Canada. PLoS Negl Trop Dis. 7(6):e2270 10.1371/journal.pntd.0002270 23818996 PMC3688548

[25] Runge M, von Keyserlingk M, Braune S, Becker D, Plenge-Bönig A, Freise JF, Pelz HJ, Esther A (2013) Distribution of rodenticide resistance and zoonotic pathogens in Norway rats in Lower Saxony and Hamburg, Germany. Pest Manag Sci. 69(3):403–408. 10.1002/ps.3369 22888034

[26] Aviat F, Blanchard B, Michel V, Blanchet B, Branger C, Hars J, Mansotte F, Brasme L, De Champs C, Bolut P, Mondot P, Faliu J, Rochereau S, Kodjo A, Andre-Fontaine G (2009) Leptospira exposure in the human environment in France: A survey in feral rodents and in fresh water. Comp Immunol Microbiol Infect Dis. 32(6):463–476. 10.1016/j.cimid.2008.05.004 18639932

[27] Trueba G, Zapata S, Madrid K, Cullen P, Haake D (2004) Cell aggregation: a mechanism of pathogenic Leptospira to survive in fresh water. Int Microbiol. 7(1):35–40. 15179605

[28] Smith DJ, Self HR (1955) Observations on the survival of Leptospira australis A in soil and water. J Hyg (Lond). 53(4):436–444. 13278528 10.1017/s0022172400000942 PMC2217981

[29] Yupiana Y, Wilson PR, Weston JF, Vallée E, Collins-Emerson JM, Benschop J, Scotland T, Heuer C (2019) Epidemiological investigation of Leptospira spp. in a dairy farming enterprise after the occurrence of three human leptospirosis cases. Zoonoses Public Health. 66(5):470–479. 10.1111/zph.12578 30942554

[30] Siqueira CC, Fraga DBM, Chagas-Junior AD, Athanazio DA, Silva MMN, Cerqueira RB, da C McBride FW, Pinna MH, Ayres MCC (2019) Seroprevalence and risk factors associated with equine leptospirosis in the metropolitan region of Salvador and Recôncavo Baiano region, Bahia state (NE Brazil). Trop Anim Health Prod. 10.1007/s11250-019-01956-5 31289965

[31] Divers TJ, Chang YF, Irby NL, Smith JL, Carter CN (2019) Leptospirosis: An important infectious disease in North American horses. Equine Vet J. 51(3):287–292. 10.1111/evj.13069 30629756

[32] Mayer-Scholl A, Hammerl JA, Schmidt S, Ulrich RG, Pfeffer M, Woll D, Scholz HC, Thomas A, Nöckler K (2014) Leptospira spp. in rodents and shrews in Germany. Int J Environ Res Public Health. 11(8):7562–7574. 10.3390/ijerph110807562 25062275 PMC4143818

[33] Turk N, Milas Z, Margaletic J, Staresina V, Slavica A, Riquelme-Sertour N, Bellenger E, Baranton G, Postic D (2003) Molecular characterization of Leptospira spp. strains isolated from small rodents in Croatia. Epidemiol Infect. 130(1):159–166. 10.1017/s0950268802008026 12613757 PMC2869950

[34] Kositanont U, Naigowit P, Imvithaya A, Singchai C, Puthavathana P (2003) Prevalence of antibodies to Leptospira serovars in rodents and shrews trapped in low and high endemic areas in Thailand. J Med Assoc Thai. 86(2):136–142. 12678151

[35] Krijger IM, Ahmed AAA, Goris MGA, Groot Koerkamp PWG, Meerburg BG (2019) Prevalence of Leptospira Infection in Rodents from Bangladesh. Int J Environ Res Public Health. 16(12). 10.3390/ijerph16122113 PMC6616592 31207905

[36] Ciceroni L, Stepan E, Pinto A, Pizzocaro P, Dettori G, Franzin L, Lupidi R, Mansueto S, Manera A, Ioli A, Marcuccio L, Grillo R, Ciarrocchi S, Cinco M (2000) Epidemiological trend of human leptospirosis in Italy between 1994 and 1996. Eur J Epidemiol. 16(1):79–86. 10.1023/a:1007658607963 10780347

[37] Jansen A, Schöneberg I, Frank C, Alpers K, Schneider T, Stark K (2005) Leptospirosis in Germany, 1962–2003. Emerg Infect Dis. 11(7):1048–1054. 10.3201/eid1107.041172 16022779 PMC3371786

[38] Hochedez P, Escher M, Decoussy H, Pasgrimaud L, Martinez R, Rosine J, Théodose R, Bourhy P, Picardeau M, Olive C, Ledrans M, Cabie A (2013) Outbreak of leptospirosis among canyoning participants, Martinique, 2011. Euro Surveill. 18(18):20472 23725775

[39] Schmalzle SA, Tabatabai A, Mazzeffi M, Matta A, Hollis A, Zubrow M, Rajagopal K, Thom K, Scalea T (2019) Recreational 'mud fever': *Leptospira interrogans* induced diffuse alveolar hemorrhage and severe acute respiratory distress syndrome in a U.S. Navy seaman following 'mud-run' in Hawaii. IDCases. 15:e00529 10.1016/j.idcr.2019.e00529 30976519 PMC6441746

[40] Muñoz-Zanzi C, Mason MR, Encina C, Astroza A, Romero A (2014) Leptospira contamination in household and environmental water in rural communities in southern Chile. Int J Environ Res Public Health. 11(7):6666–6680. 10.3390/ijerph110706666 24972030 PMC4113836

[41] Ganoza CA, Matthias MA, Collins-Richards D, Brouwer KC, Cunningham CB, Segura ER, Gilman RH, Gotuzzo E, Vinetz JM (2006) Determining risk for severe leptospirosis by molecular analysis of environmental surface waters for pathogenic Leptospira. PLoS Med. 3(8):e308 10.1371/journal.pmed.0030308 16933963 PMC1551915

[42] Casanovas-Massana A, Costa F, Riediger IN, Cunha M, de Oliveira D, Mota DC, Sousa E, Querino VA, Nery N Jr, Reis MG, Wunder EA Jr, Diggle PJ, Ko AI (2018) Spatial and temporal dynamics of pathogenic Leptospira in surface waters from the urban slum environment. Water Res. 130:176–184. 10.1016/j.watres.2017.11.068 29220718 PMC5767135

