# Supplementary material for: Evidence of Leptospiral Presence in the Cumberland Gap Region
Source: PLoS Negl Trop Dis. 2019 Dec 26;13(12):e0007990. doi: 10.1371/journal.pntd.0007990 (PMC6952108; doi:10.1371/journal.pntd.0007990)
Supplement: S1 Table — (PPTX) [file pntd.0007990.s001.pptx]

## Slide 1
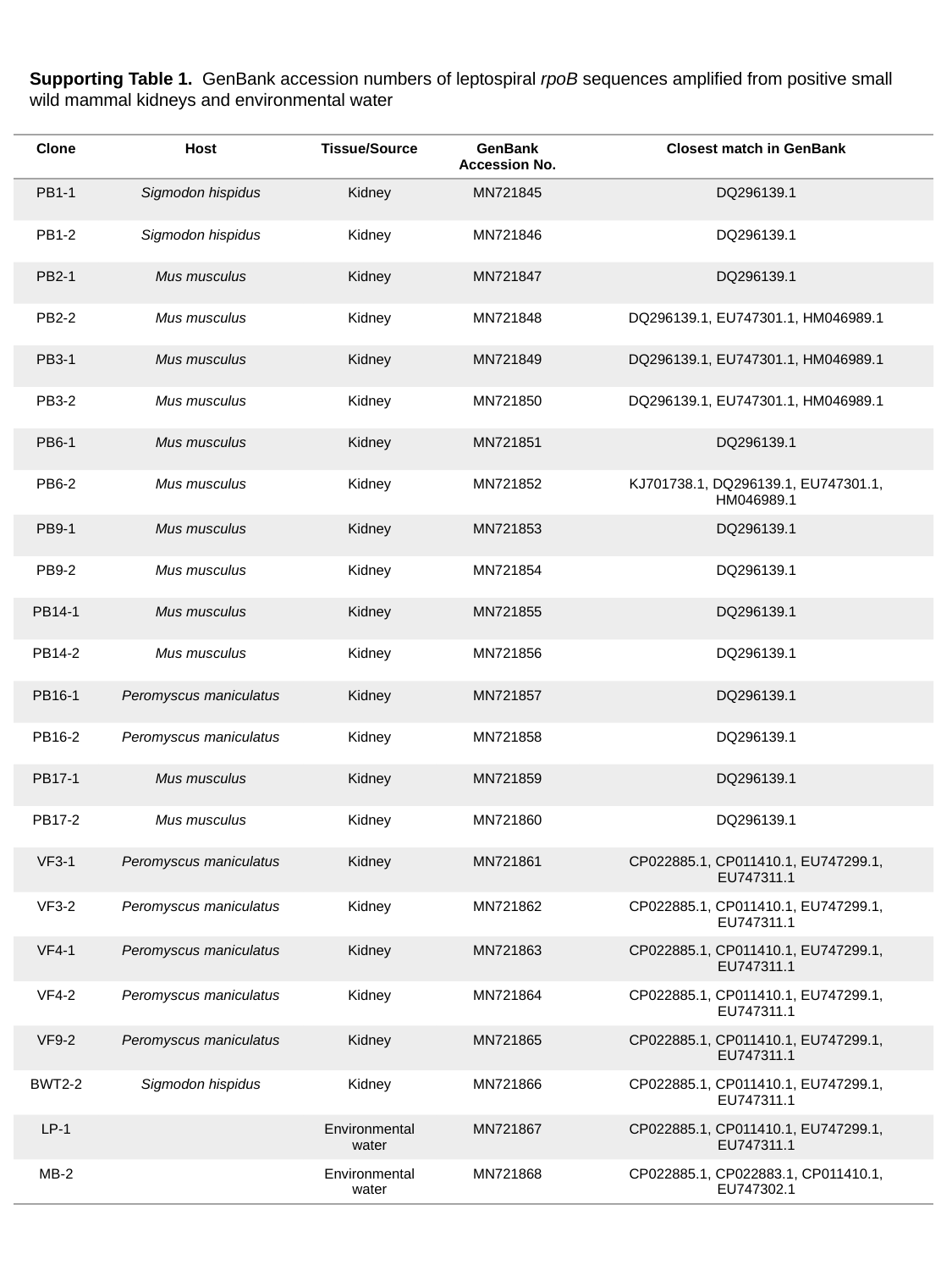

Supporting Table 1. GenBank accession numbers of leptospiral rpoB sequences amplified from positive small wild mammal kidneys and environmental water
| Clone | Host | Tissue/Source | GenBank Accession No. | Closest match in GenBank |
| --- | --- | --- | --- | --- |
| PB1-1 | Sigmodon hispidus | Kidney | MN721845 | DQ296139.1 |
| PB1-2 | Sigmodon hispidus | Kidney | MN721846 | DQ296139.1 |
| PB2-1 | Mus musculus | Kidney | MN721847 | DQ296139.1 |
| PB2-2 | Mus musculus | Kidney | MN721848 | DQ296139.1, EU747301.1, HM046989.1 |
| PB3-1 | Mus musculus | Kidney | MN721849 | DQ296139.1, EU747301.1, HM046989.1 |
| PB3-2 | Mus musculus | Kidney | MN721850 | DQ296139.1, EU747301.1, HM046989.1 |
| PB6-1 | Mus musculus | Kidney | MN721851 | DQ296139.1 |
| PB6-2 | Mus musculus | Kidney | MN721852 | KJ701738.1, DQ296139.1, EU747301.1, HM046989.1 |
| PB9-1 | Mus musculus | Kidney | MN721853 | DQ296139.1 |
| PB9-2 | Mus musculus | Kidney | MN721854 | DQ296139.1 |
| PB14-1 | Mus musculus | Kidney | MN721855 | DQ296139.1 |
| PB14-2 | Mus musculus | Kidney | MN721856 | DQ296139.1 |
| PB16-1 | Peromyscus maniculatus | Kidney | MN721857 | DQ296139.1 |
| PB16-2 | Peromyscus maniculatus | Kidney | MN721858 | DQ296139.1 |
| PB17-1 | Mus musculus | Kidney | MN721859 | DQ296139.1 |
| PB17-2 | Mus musculus | Kidney | MN721860 | DQ296139.1 |
| VF3-1 | Peromyscus maniculatus | Kidney | MN721861 | CP022885.1, CP011410.1, EU747299.1, EU747311.1 |
| VF3-2 | Peromyscus maniculatus | Kidney | MN721862 | CP022885.1, CP011410.1, EU747299.1, EU747311.1 |
| VF4-1 | Peromyscus maniculatus | Kidney | MN721863 | CP022885.1, CP011410.1, EU747299.1, EU747311.1 |
| VF4-2 | Peromyscus maniculatus | Kidney | MN721864 | CP022885.1, CP011410.1, EU747299.1, EU747311.1 |
| VF9-2 | Peromyscus maniculatus | Kidney | MN721865 | CP022885.1, CP011410.1, EU747299.1, EU747311.1 |
| BWT2-2 | Sigmodon hispidus | Kidney | MN721866 | CP022885.1, CP011410.1, EU747299.1, EU747311.1 |
| LP-1 | | Environmental water | MN721867 | CP022885.1, CP011410.1, EU747299.1, EU747311.1 |
| MB-2 | | Environmental water | MN721868 | CP022885.1, CP022883.1, CP011410.1, EU747302.1 |
